# Giant magnetoelectric coupling observed at high frequency in NiFe_2_O_4_–BaTiO_3_ particulate composite

**DOI:** 10.1039/d0ra05782g

**Published:** 2020-07-21

**Authors:** Zhenhua Shi, Jing Zhang, Daqiang Gao, Zhonghua Zhu, Zhaolong Yang, Zhipeng Zhang

**Affiliations:** School of Science, Xi'an Technological University Xi'an 710021 People's Republic of China shizhenhua@xatu.edu.cn; Key Laboratory for Magnetism and Magnetic Materials of MOE, Lanzhou University Lanzhou 730000 People's Republic of China

## Abstract

A giant magnetoelectric voltage coupling coefficient without direct current magnetic field observed in NiFe_2_O_4_–BaTiO_3_ particulate composite is reported. The particulate composite was obtained by combining hydrothermal and sol–gel method, and was studied for their crystallographic structure, morphology, magnetic, dielectric and magnetoelectric properties. Results of Mössbauer spectra demonstrated the presence of interface phase in particulate composite, where the changes of the magnetic properties in composite compared to the pure NiFe_2_O_4_ also confirmed this. The particulate composite exhibits remarkable magnetoelectric effect through both static measurement and dynamic measurement. The special magnetoelectric property of the particulate composite is beneficial for applications in high frequency devices.

## Abstract 2

A giant magnetoelectric voltage coupling coefficient without direct current magnetic field observed in NiFe_2_O_4_–BaTiO_3_ particulate composite is reported.

## Graphical abstract

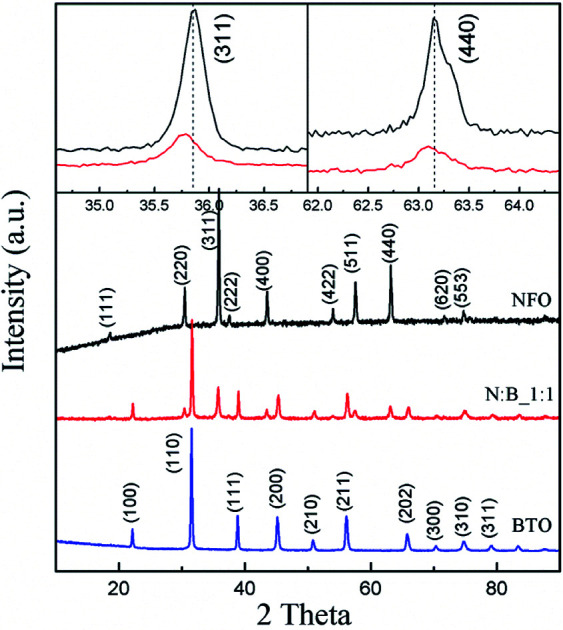

## Introduction

Multiferroics in which two or more ‘ferroic’ properties such as ferroelectricity, ferromagnetism and ferroelasticity can coexist and be coupled to each other have attracted much attention in recent years.^1–4^ Because of the magnetoelectric (ME) coupling effect, multiferroics display novel physical phenomena^5–7^ and can be used as magnetic probes, transducers, novel actuators, sensors and magnetic field tunable microwave devices.^8–12^ Generally, ME coupling is characterized by an electric polarization response to an applied magnetic field or conversely a magnetic polarization response to an applied electric field. In single-phase multiferroic materials, the ME effect is low and the ME coupling usually occurs under extreme conditions, in particular, very low temperatures^13–15^ and the applications have been limited. However, multiferroic composites can overcome these limits and exhibit excellent ME effect above room temperature (RT), so they have attracted particular research interest.^16–18^ Multiferroic composites are usually made by combining ferroelectric and magnetic phases, and could have various connectivity schemes, but the common connectivity schemes are 0–3–type particulate composites, 2–2–type laminate composites, and 1–3–type fiber composites with fibers of one phase embedded in the matrix of another phase.^2^ The ME sensitivity of such 0–3 type and pseudo 2–2 type composites is about 10 mV cm^−1^ Oe^−1^ and 100 mV cm^−1^ Oe^−1^ order of magnitude, respectively.^19,20^ For 1–3 ME composite, Ma *et al.* reported that for arrays of PZT rods embedded in Terfenol-D/Epoxy matrix, the ME voltage coupling coefficient reaches 500 mV cm^−1^ Oe^−1^.^21^ Generally, the ME effect in multiferroic composites is induced by the interface between two phases,^7,22,23^ and is dependent on the interface scheme. Furthermore, large ME effect need an external dc magnetic field. Most recently, researchers have turned their interest to multiferroic nanocomposites,^24–27^ because of the demand for high-density electronic components with high performance, multifunctionality, smaller size and low cost. In nanoscale, the composites hold high contact area between the components and the interfaces play a key role in modulating the effective material properties, furthermore, the nanocomposites exhibit novel ME phenomena.^5,24,28^

It is easy to synthesize 0–3 type structured composites. Since the interface is of great significance for composites,^7,29^ it is inevitable that the property of interface is able to influence the ME effect. A kind of interface which is benefit to ME coupling effect should be organized. The ferroelectric and magnetic phases must be are tightly combined, and there is no structure phase transition at interface. This is good for stress effects. BaTiO_3_ (BTO) which has good chemical stability, high dielectric constant and low loss in a wide frequency range is a typical lead-free ferroelectric material. NiFe_2_O_4_ (NFO) is a soft magnetic material and displays high permeability and low magnetic anisotropy.^30–32^ This promotes a high value of coupling for NFO-based multiferroic composites materials. On the other hand, it also has a much larger resistivity and NFO-based multiferroic composite materials can be used in high frequency. In this paper, NFO–BTO particulate composite (PC) has been successfully synthesized. The structure, morphology, magnetic, dielectric and ME characterization of the samples were systemically investigated. Importantly, the presence of interface phase is demonstrated by Mössbauer spectra and magnetic properties analysis in the PC. Simultaneously, large ME coupling effect has been obtained at high frequency. These properties imply that the PC shows promising potential in high frequency applications.

## Experimental section

### Materials

Fe(NO_3_)_3_·9H_2_O (98%), KOH (99%) and Barium chloride (BaCl_2_, 99%) were purchased from Alfa Aesar. Ammonia (28%), citric acid (99%), acetylacetone (C_5_H_8_O_2_, 99%), tetrabutyl titanate (C_16_H_36_O_4_Ti, 98%) and Ni(NO_3_)_2_·6H_2_O (98%) were acquired from Shanghai Hushi. All the chemical regents in the experimental process were used as received without further purification.

### Synthesis of NFO–BTO particulate composite

The NFO–BTO PC was synthesized by a two-step approach: preparing of monodisperse BTO particles and then dispersing them in the NFO matrix. Firstly, 0.01 M barium chloride (BaCl_2_) was dissolved in 30 ml of distilled water and 0.01 M tetrabutyl titanate (C_16_H_36_O_4_Ti) was dissolved in 4 ml of acetylacetone (C_5_H_8_O_2_) under mechanical stirring. After dissolution, the solution containing titanium was dropped into the aqueous solution of BaCl_2_ with stirring for a few minutes, Then KOH solution was slowly added to coprecipitate metal ions and adjust the pH to 14. After vigorously stirring for 30 min, the suspension solution was transferred into the autoclave filled up to 4/5 of its capacity. The hydrothermal treatment was performed at 160 °C for 48 h. Finally, the products were washed with deionized water and absolute ethanol several times to remove any impurities, and were dried at 80 °C to obtain BTO particles. Secondly, 0.005 M Ni(NO_3_)_2_·6H_2_O, 0.01 M Fe(NO_3_)_3_·9H_2_O and 0.015 M citric acid were dissolved in 50 ml de-ionized water. The molar ratio of metal ions to citric acid was 1 : 1. A small amount of ammonia was added to the solution to adjust the pH value at about 7 with continuously stirring. Then, the dissolved solution was stirred for 5 h in an oil bath at 80 °C. Subsequently, 0.005 M BTO particles were added into the solution with continuing stirring at 80 °C until dry, and the mixture was cured in an oven to form the precursor at 140 °C. The precursor was pre-annealed at 400 °C for 2 h, and then calcined at 700 °C for 2 h in the air. The PC contain NFO and BTO in a molar ratio of 1 : 1 was denoted as N : B_1 : 1. For comparison, pure BTO and NFO powders were prepared under the same conditions.

### Characterization

The X-ray diffraction (XRD, X′ Pert PRO PHILIPS with Cu Kα radiation) was employed to study the structure of the samples. The morphologies of the samples were characterized by the transmission electron microscope (TEM; Tecnai TMG2F30, FEI, Hillsboro, OR, USA) equipped with energy-dispersive X-ray spectrum (EDS). The measurements of magnetic properties were made using the vibrating sample magnetometer (VSM; micorsense, EV9), and a Quantum Design MPMS magnetometer based on a superconducting quantum interference device (SQUID; San Diego, CA, USA). Mössbauer measurements were performed using conventional constant acceleration type spectrometer in transmission geometry. The gamma-ray source is 25 mCi57Co in Palladium matrix and the driver velocity was calibrated using an α-Fe foil. The dielectric properties of the samples were measured using an Agilent 4092A precision impedance analyzer in the temperature range from 230 to 400 K.

## Results and discussions

**Fig. 1:**
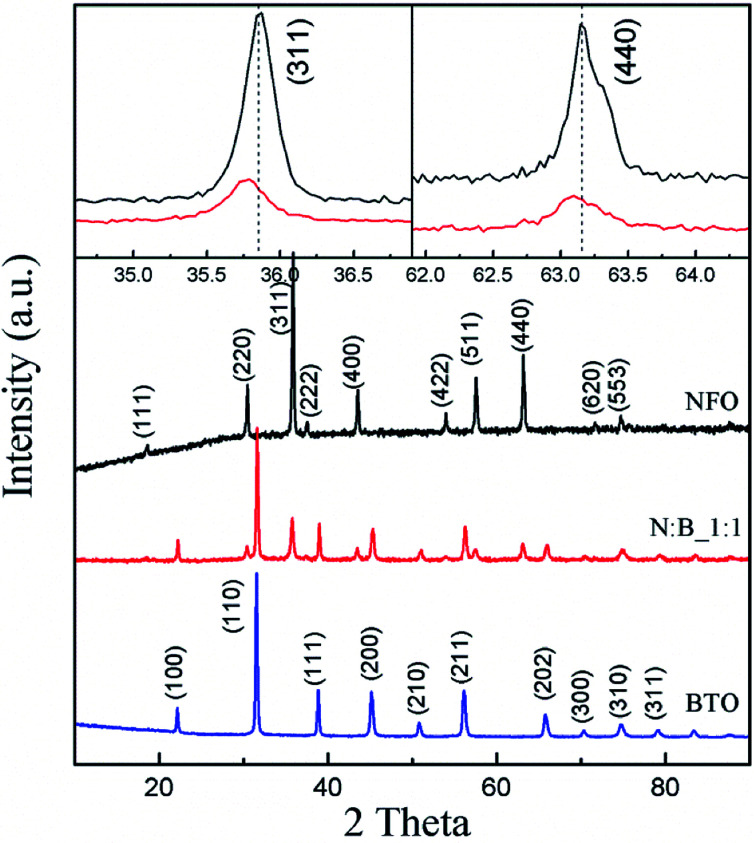
XRD patterns of samples. Inset is an enlarged view of (311) and (440) diffraction peaks for NFO and PC.

**Fig. 2:**
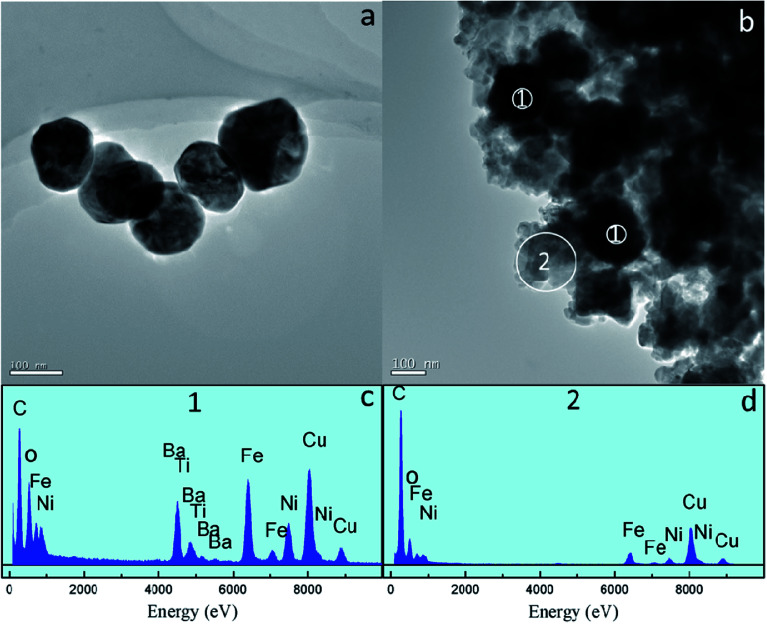
(a) TEM image of BTO, (b) TEM image of PC (c and d) EDS spectra acquired at positions 1 and 2 in (b).

The XRD patterns of the samples are shown in Fig. 1. The pure perovskite BTO phase and spinel NFO phase are obtained. In PC, only the perovskite and spinel phases corresponding to BTO and NFO are observed, respectively. No trace of other phases has been found. Further analysis results indicate that BTO exhibits a tetragonal structure because the (200) and (211) diffractions are broader than the (111) peak, which means that BTO has a polar structure in PC.^33,34^ However, peaks corresponding to the perovskite phase are not split as they should be for bulk BTO at RT, this indicates that the tetragonal distortion of the unit cell is small. Fig. 2(a and b) shows a typical TEM image of the hydrothermally synthesized BTO particles and the PC sample. Results indicate that the average particle size of BTO is about 130 nm. We have investigated the spatial distribution of the two phases on the N : B_1 : 1 (Fig. 2(b)). It is clearly seen that the BTO particles uniformly dispersed in NFO matrix and the spatial distribution of the two phases is well supported by the EDS analysis (Fig. 2(c and d)): Fe, Ni, Ba and Ti elements are detected in the region marked 1, whereas Fe and Ni dominate the region marked 2. The peak of Cu is originating from the TEM sample grid. This spatial distribution of the two phases is good to increase the interface-to-volume ratio, perhaps this is also beneficial to ME effect.

**Fig. 3:**
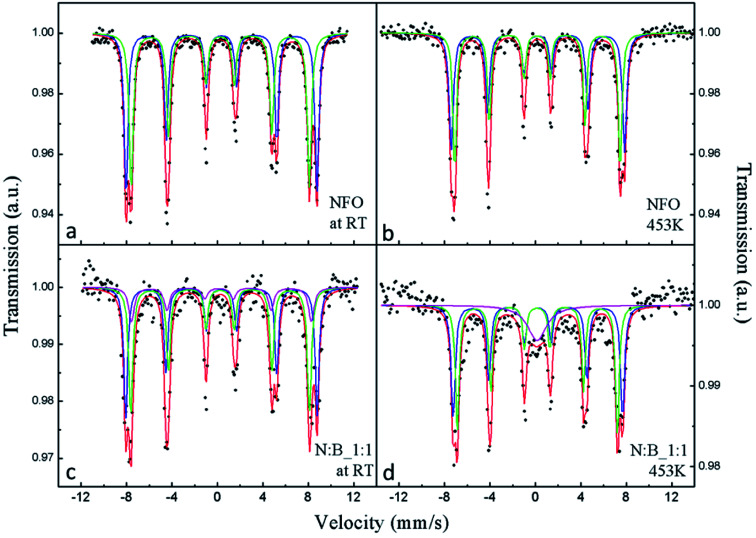
^57^Fe Mössbauer spectra of NFO at (a) RT, (b) 453 K and PC at (c) RT, (d) 453 K.

To investigate the magnetic component parts of the PC, ^57^Fe Mössbauer measurement at different temperatures are employed and the results are shown in Fig. 3(a–d). The hyperfine parameters obtained by fitting are listed in Table 1. The Mössbauer spectra of NFO (Fig. 3(a and b)) can be well fitted with two different sets of magnetically split sextets corresponding to iron ions in tetrahedral site (T) and octahedral site (O). The magnetic hyperfine field extracted by fitting the measured spectra of NFO at RT is 52.1 and 48.6 T corresponding to O site and T site, respectively, which is consistent with previous reports.^35,36^ At 453 K, the magnetic hyperfine field of NFO is slightly lower than that observed at RT, and it also shows large magnetic hyperfine field indicating that NFO exhibits a magnetic behavior. The spectra (Fig. 3(c and d)) of PC can be fitted with the superposition of three magnetic sextets. In addition to the above mentioned two sextets, the other sextet (3^rd^ sextet) is probably connected with the iron ions at the interface between NFO and BTO particles. Interface layers can differ in chemical composition in comparison with the interior part of the particles. Therefore, we ascribed the 3^rd^ sextet to the iron atoms in interfaces of the particles. The 3^rd^ sextet has an obvious magnetic hyperfine field in Fig. 3(c), and this reveals that all NFO exhibits a magnetic behavior at RT. However, when the temperature raise up to 453 K, the overall splitting of the outer emission lines, that measures the magnetic hyperfine field at the nucleus, drastically disappear for the 3^rd^ sextet, revealing the presence of a magnetic phase transition. The above results indicate that a part of NFO for PC shows special magnetic property compared with the purely NFO, and should be at the interface between NFO and BTO. From the inset of Fig. 1, the (311) and (440) diffraction peaks of PC shift towards lower scattering angle as compared to that of NFO, this means the unit cell of NFO in PC expand thanks to the substitution of barium and titanium ions with larger ionic radius in the NFO lattice. This also indicates the magnetic property of NFO at interface is different from purely NFO due to the ion diffusion. The relative spectral contributions of the 3^rd^ sextet to overall NFO spectral profile is approximately 15% at both temperatures, meaning the interface phase is approximately 15% of NFO in the PC and does not increase or decrease.

**Table 1:** Hyperfine parameters of samples. *I* is corresponding to the interface

		IS (mm s^−1^)	QS (mm s^−1^)	H (T)	LW (mm s^−1^)	W (%)
NFO	O	0.36	−0.00	52.1	0.40	49.2
	T	0.24	0.01	48.6	0.44	50.8
PC	O	0.35	0.00	52.2	0.40	41.5
	T	0.24	0.00	48.7	0.43	43.1
	I	0.24	0.21	49.1	0.57	15.4
NFO 453 K	O	0.21	0.00	47.3	0.43	49.5
	T	0.16	0.02	45.1	0.38	50.5
PC 453 K	O	0.21	0.00	46.2	0.43	41.7
	T	0.16	0.01	43.7	0.38	43.1
	I	0.15	—	—	2.01	15.2

**Fig. 4:**
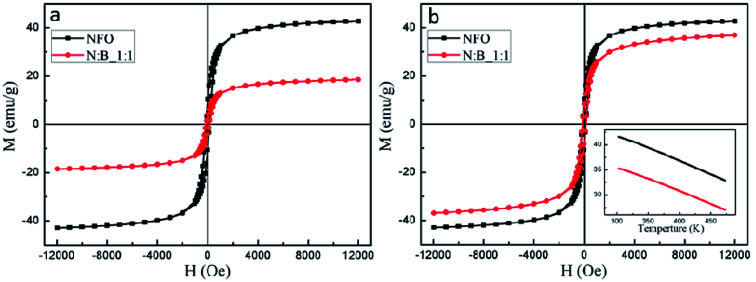
(a) The *M*–*H* loops for samples; (b) the calculated *M*–*H* loops for samples, inset: magnetization *versus* temperature curve for samples measured at 8000 Oe.

The static magnetic properties of the NFO and the PC were studied by VSM. The magnetization hysteresis (*M*–*H*) loops for NFO and the PC at RT with both ±1.2 T magnetic field are displayed in Fig. 4(a). It is found that the value of saturation magnetization (*M*_s_) is 42.6 emu g^−1^ for NFO and 18.4 emu g^−1^ for N : B_1 : 1, respectively. Generally, the *M*_s_ of NFO particles is lower than that of bulk form (56 emu g^−1^),^37,38^ which can be attributed to the small grain size and surface effects.^39^ The *M*_s_ of the PC is significantly lower than that of NFO, due to the nonmagnetic BTO. The value of magnetization (*M*) for NFO in the PC can be calculated using *M* = *I*/(*mw*), in which *I* is the signal intensity of the VSM, *m* is the mass of the PC and *w* is the mass fraction of the NFO in the PC. The results are shown in Fig. 4(b). As shown in the image, the *M*_s_ of NFO in the PC is 36.8 emu g^−1^ and significantly lower than purely NFO (∼14% of *M*_s_ reduces), which is attributed to the interface phase. According to the results of Mössbauer spectra, the NFO at interface accounted for 15% of overall NFO in PC, moreover, this part of NFO shows a magnetic phase transition in the temperature range of from RT to 453 K and exhibits nonmagnetic at 453 K. However, the curves (inset in Fig. 4(b)) of magnetization as a function of temperature for NFO and PC with the magnetic field at 8000 Oe show the same trends. In addition to the gradually decreasing of magnetization with increasing temperature, no distinct change of the magnetization for PC has been observed. Meanwhile, the difference of magnetization between NFO and PC remains constant in the test temperature range. This means that the difference of magnetization does not change, whether the interface phase exhibits magnetic hyperfine field or not. This would imply that the interface phase shows no net magnetic moment in the temperature range from RT to 453 K. According to Mössbauer spectra and static magnetic properties of the samples, perhaps the magnetic order state of the NFO at interface is an antiferromagnetic one. The *M*_s_ of PC reduce thanks to the presence of a nonmagnetic perovskite phase and the interface effects.

**Fig. 5:**
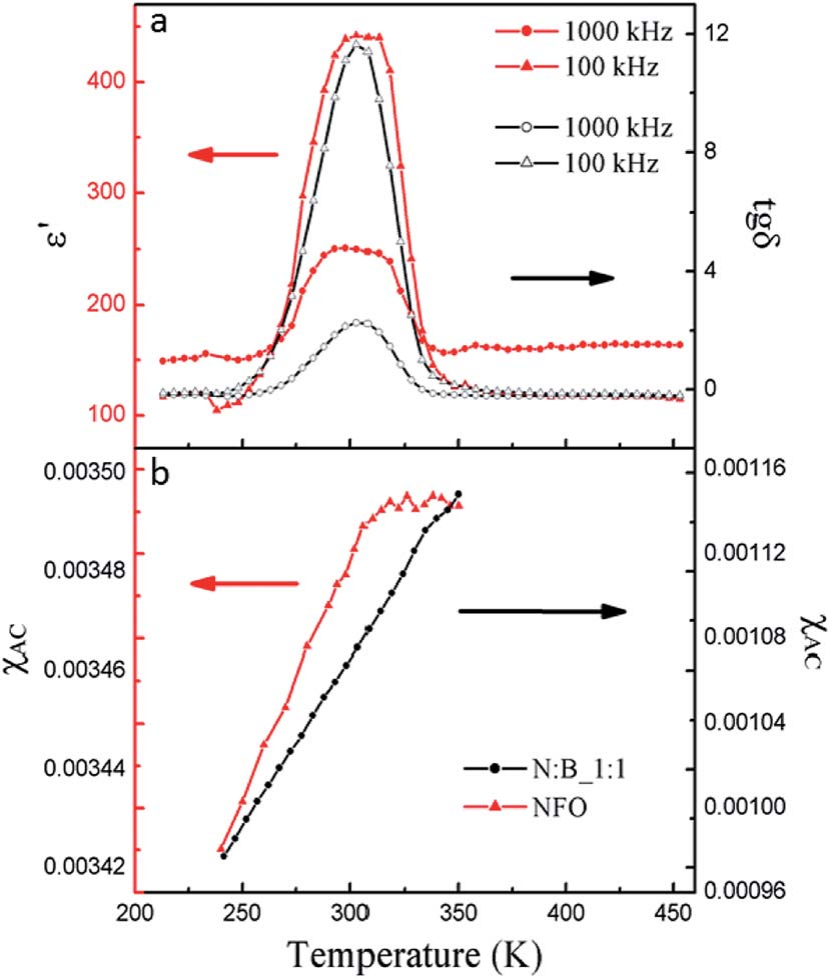
(a) Temperature dependence of the dielectric constant (*ε*′) and dielectric loss (tg*δ*) of PC with a frequency of 100 and 1000 kHz; (b) temperature dependence of *χ*_AC_ for NFO and PC.

The temperature dependence of dielectric permittivity and dielectric loss at 100 and 1000 kHz obtained from PC specimen are depicted in Fig. 5(a). The dielectric permittivity at these high frequencies has only an intrinsic contribution from PC specimen, free from the space-charge contributions coming from electrode and grain boundaries.^40^ The results reveal clearly the presence of broad dielectric peaks occurring around 300 K independent of the frequency. It is well known that a ferroelectric phase transition can make the dielectric constant divergent, so the peaks of dielectric permittivity curves are evident for the ferroelectric transition of the PC. The change of the BTO ferroelectric transition is due to the size effect on phase transition.^41,42^ The peaks of the curves are very broadening with a temperature range from 280 K to 330 K, which is due to the heterogeneity of the local structure of PC and the size of BTO. Meanwhile, the PC shows a quite low dielectric loss. The temperature dependence of the alternating current (ac) magnetic susceptibility (*χ*_AC_) at 1000 Hz is presented in Fig. 5(b). An anomaly of ac magnetic susceptibility appears at around 300 K which occurs in the temperature range of the ferroelectric transition for PC compare to the purely NFO. These results show that the ferroelectric phase has a significant effect on magnetic property of NFO and indicate that there is strong coupling between magnetic phase and ferroelectric phase.

**Fig. 6:**
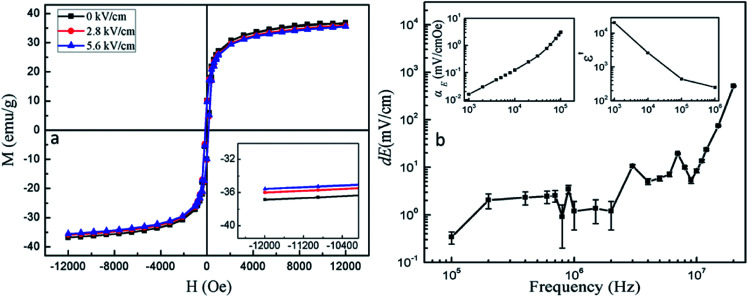
(a) RT *M*–*H* loop of PC under different E_dc_ (zero, 2.8 and 5.6 kV cm^−1^), inset: the partially enlarged *M*–*H* loops; (b) the d*E* of PC as a function of the frequency range is 10^5^ to 10^7^ Hz, upper left inset in (b): the *α*_E_ of PC as a function of the frequency range is 10^3^ to 10^5^ Hz; upper right inset (b): frequency dependence of the dielectric constant of PC.

The electric-field controlled ferromagnetism, which is usually defined as converse ME effect, has been investigated by measuring the RT *M*–*H* loops of PC with direct current (dc) electric field (*E*) perpendicular to the magnetic field (*H*). An obvious reduction of *M*_s_ with the increase of *E* is observed in Fig. 6(a). The change of *M*_s_ reaches up to 3.4% by applying an electric field of 5.6 kV cm^−1^. This can be attributed to the presence of elongation along the *E* direction for ferroelectric phase when *E* is applied to the PC. And this elongation produces compressive stress along *E* direction which is perpendicular to the *H* direction and tensile stress along H direction in the magnetic phase. Furthermore, NFO shows negative magnetostriction according to the previous reports.^43,44^ Therefore, the magnetization decreases in *H* direction that is also the measuring direction of magnetization. In order to further identify the real ME coupling in PC, a dynamic technique has been employed. To measure the ME voltage coefficients, an ac magnetic field *H*_ac_ superimposed on a dc magnetic field *H*_dc_ was applied on the sample and the induced voltage (d*V*) was measured using a lock-in amplifier or an oscilloscope.^45,46^ The ME voltage coupling coefficient is defined as *α*_E_ = dE/d*H*, in which d*E* = d*V*/*t* (*t* is the sample thickness), and d*H* is the *H*_ac_. The *α*_E_ and d*E* was measured without *H*_dc_ at RT over a frequency range of 1 < *f* < 10^5^ Hz (upper left inset in Fig. 6(b)) and 10^5^ < *f* < 2 × 10^7^ Hz (Fig. 6(b)), respectively. Although the value of *H*_ac_ at high frequency is hard to measure, it decreases with the increase of frequency, because the impedance of coil increase with the frequency increasing, and the input power is constant. When the frequency is higher than 10^5^ Hz, the value of *H*_ac_ is lower than 0.11 Oe which is measured at 10^5^ Hz. Therefore, the *α*_E_ shows larger value in high frequency range, and it is more than 5 V cm^−1^ Oe^−1^ at 20 MHz. Results indicate that the *α*_E_ continuously increases as frequency increases. This is mainly attributed to the decreasing dielectric constant of the PC with increasing frequency (upper right inset in Fig. 6(b)). Because the *α*_E_ = d*E*/d*H* ≈ *α*/*ε*_0_*ε*^47,48^ (*α* is the linear ME coupling coefficient, *ε*_0_ is the free space permittivity, *ε* is the relative permittivity) is inversely proportional to dielectric constant in our case, moreover, the *α*_E_ shows large value without *H*_dc_, this is different from previous reports^49,50^ in which the composites show *α*_E_ often need *H*_dc_. These special ME effects in PC may be produced by the interface phase between ferroelectricity phase and ferromagnetism phase. This special property is beneficial for application in high frequency devices. Smaller composites are benefit for increasing the interface-to-volume ratio. It is meaningful to investigate the interface and ME effect of smaller composites (less than 100 nm). J. P. Zhou *et al.* reported that NiFe_2_O_4_@BaTiO_3_ composite with an average grain size of 200 nm were prepared by the hydrothermal method.^19^ M. Reaz *et al.* reported that BaTiO_3_/iron oxide core–shell nanoparticles with an average grain size of 100 nm were prepared by the physiochemical synthesis process.^51^ In our best knowledge, it is difficult to reduce the composites particle size, this work will be done in the future.

## Conclusion

In summary, the NFO–BTO PC was obtained by the two-step approach, as confirmed by XRD, TEM and EDX analysis. The interface phase has been observed in the PC, and the *M*_s_ of NFO in PC significantly lower than purely NFO, thanks to the presence of interface phase. The ac magnetic susceptibility shows an abnormal around the ferroelectric phase transition temperature. The PC exhibits remarkable ME effect through both static measurement and dynamic measurement. Notably, the PC exhibits giant ME voltage coupling coefficient without dc magnetic field, this is beneficial for application in high frequency devices.

## Conflicts of interest

There are no conflicts to declare.

## Supplementary Material
