# Correction: Giant magnetoelectric coupling observed at high frequency in NiFe_2_O_4_–BaTiO_3_ particulate composite

**DOI:** 10.1039/d2ra90101c

**Published:** 2022-10-10

**Authors:** Zhenhua Shi, Jing Zhang, Daqiang Gao, Zhonghua Zhu, Zhaolong Yang, Zhipeng Zhang

**Affiliations:** School of Science, Xi'an Technological University Xi'an 710021 People's Republic of China shizhenhua@xatu.edu.cn; Key Laboratory for Magnetism and Magnetic Materials of MOE, Lanzhou University Lanzhou 730000 People's Republic of China

## Abstract

Correction for ‘Giant magnetoelectric coupling observed at high frequency in NiFe_2_O_4_–BaTiO_3_ particulate composite’ by Zhenhua Shi *et al.*, *RSC Adv.*, 2020, **10**, 27242–27248, https://doi.org/10.1039/D0RA05782G.

The authors regret that an incorrect grant number was shown in the acknowledgements section of the published article. The corrected section should read:

We acknowledge the financial support from National Natural Science Foundation of China (11904275).

The Royal Society of Chemistry apologises for these errors and any consequent inconvenience to authors and readers.

## Supplementary Material

